# Toward the Thermoelectric ZT Limit via the Quantification of Interface‐Driven Carrier Sorting

**DOI:** 10.1002/smll.202505325

**Published:** 2025-07-25

**Authors:** Xiwen Zhang, Yi‐ming Zhao, Liang Ma, Zhensen Chen, Yunfei Chen, Jinlan Wang, Lei Shen

**Affiliations:** ^1^ School of Mechanical Engineering Southeast University Nanjing 211189 China; ^2^ Key Laboratory of Quantum Materials and Devices of Ministry of Education School of Physics Southeast University Nanjing 211189 China; ^3^ Department of Mechanical Engineering National University of Singapore Singapore 117575 Singapore

**Keywords:** carrier sorting, heterostructures, interfacial transport, thermoelectric optimization

## Abstract

Interfacial engineering is a promising strategy to enhance thermoelectric performance, but identifying and optimizing the interfacial carrier transport mechanisms required to approach the theoretical ZT limit remains challenging. Here, a unified, quantitative framework is presented to describe and correlate cross‐interface transport with thermoelectric properties in heterostructures. Using SnSe/GeSe superlattices as a model, an effective interfacial energy‐sorting potential (*Φ*_eff_) is introduced, defined as *Φ*_eff_ = Δ*E* − *δ*, where Δ*E* is the valence band offset and *δ* accounts for interface‐induced barrier softening. This enables the direct extraction of extrinsic thermoelectric contributions, including Δ*S* (Seebeck coefficient), Δ*σ* (electrical conductivity), and Δ*P* (power factor). An inverse relationship between Δ*S* and Δ*σ* is revealed, resulting in a nonmonotonic dependence of Δ*P* on *Φ*_eff_. An analytical volcano plot identifies an optimal Δ*E* of≈0.48 eV for maximizing Δ*P*. At this condition, a four‐layer SnSe/GeSe structure is predicted to achieve a ZT of 2.01, which is remarkable among reported nanoscale thermoelectric materials. This work offers a generalizable strategy for quantifying interface‐governed transport and provides valuable insights into the design of high‐performance nano‐thermoelectric materials and devices.

## Graphical abstract

This study reveals the critical role of interface‐driven carrier sorting (IDCS) in enhancing thermoelectric performance and establishes a quantitative framework to elucidate the relationship between IDCS and enhancement in transport properties. A volcano‐shaped extrinsic power factor diagram is constructed, offering predictive guidance for interface‐induced performance optimization, enabling peak thermoelectric performance in heterostructures and advancing next‐generation thermoelectric technologies.

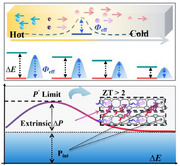

## 1 Introduction

The rising global energy demand and mounting environmental concerns have prompted the scientific community to explore efficient and sustainable energy conversion technologies. Thermoelectric materials, which convert the waste heat directly into electricity without pollution and noise, have emerged as a key focus of energy research.^[^ ^1^ ^]^ The dimensionless thermoelectric figure‐of‐merit (*ZT* = *σS* ^2^ *T*/*κ*) and power factor (*P* = *σS* ^2^) are the material‐level performance scales for thermoelectric conversion efficiency and output power factor.^[^ ^2^ ^]^ Here, *S*, *σ*, *κ*, and *T* are the Seebeck coefficient, electrical conductivity, total thermal conductivity composed of lattice thermal conductivity (*κ*_l_) and electronic thermal conductivity (*κ*_e_), and temperature, respectively. Low‐dimensional materials, particularly two ‐ dimensional (2D) systems, hold great potential for achieving high *ZT* values due to quantum confinement effects, which optimize band structure and enhance carrier transport. Their inherent mechanical flexibility also makes them promising for next‐generation flexible electronics.^[^ ^3^ ^]^ However, in low‐dimensional materials, quantum confinement and enhanced electron‐phonon interactions increase the interdependence of thermoelectric parameters, making independent tuning more difficult compared to bulk materials.^[^ ^4^ ^]^ As a result, while *ZT* values exceeding 2 have been achieved in many bulk thermoelectric materials, realizing such high *ZT* in nanoscale systems remains a major challenge.^[^ ^5^ ^]^

Recent studies have shown that introducing heterointerfaces into thermoelectric materials, including through heterostructures or composites, can reduce lattice thermal conductivity by enhancing phonon scattering and potentially improve the power factor through interfacial modulation of carrier transport.^[^ ^6^ ^]^ This strategy offers promising opportunities to achieve high *ZT* values, especially in low‐dimensional systems. However, experimental results have been inconsistent. While some systems exhibit improved thermoelectric performance after interface introduction, others demonstrate negligible or even adverse effects.^[^ ^6^, ^7^ ^]^ These inconsistencies suggest that constructing interfaces alone does not guarantee performance enhancement, and that the underlying transport mechanisms must be better understood. Among the factors related to interfaces, interlayer interactions usually have a relatively minor impact and can be described using existing physical models.^[^ ^8^ ^]^ In contrast, carrier transport across interfaces plays a much more decisive role in determining electrical properties. Although previous studies have investigated interface‐related effects, most of them focus on polycrystalline grain boundaries or doping‐induced transport features, which do not apply well to well‐defined heterostructures.^[^ ^9^ ^]^ Moreover, conventional first‐principles thermoelectric models are limited in their ability to capture carrier transport effects induced by heterointerfaces. This limitation creates a persistent gap between theoretical predictions and experimental observations, which hinders the rational design and optimization of heterostructure‐based thermoelectric materials.^[^ ^10^ ^]^ Therefore, developing a systematic and quantitative framework to describe interface‐driven carrier transport is essential, yet currently limited.

In this study, we unveil the heterointerface energy‐sorting mechanism which extends classical energy filtering by incorporating partial quantum tunneling and interface‐induced smoothing effects using the SnSe/GeSe superlattice platform and establish a quantitative framework to tune the effective energy‐sorting potential (*Φ*_eff_) for optimizing thermoelectric transport in heterostructures. Through a comparative transport analysis along the interfacial direction in both heterostructures and homostructures, we elucidate the crucial role of interface engineering in enhancing energy‐sorting carrier transport for improved thermoelectric performance. Our calculations reveal a strong correlation between the interface‐induced extrinsic thermoelectric parameters (Δ*S*, Δ*σ*, and Δ*P*) and the effective potential *Φ*_eff_, defined as *Φ*_eff_ = Δ*E* − *δ*. Here, Δ*E* represents the valence band offset, while the correction term *δ* accounts for barrier softening effects arising from partial quantum tunneling and interface‐induced smoothing. Leveraging these insights, we develop a physics‐based analytical model and introduce a volcano diagram: Δ*P* = −*a*(Δ*E* − 0.48)^2^ + *b*, where *a* and *b* are positive constants. This volcano diagram predicts that Δ*P* reaches its maximum at an optimal Δ*E* of 0.48 eV, identifying a critical *Φ*_eff_ threshold that governs carrier‐sorting transport for thermoelectric optimization. Further, cross‐validation with experimental data from various heterostructures confirms the broad applicability of the volcano diagram. Under the optimal Δ*E*, a four‐layer SnSe/GeSe heterostructure achieves a *ZT* of 2.01, reaching a highly competitive level in nanoscale thermoelectric performance. Overall, this work presents a broadly applicable and quantitatively predictive framework that links interface‐induced transport effects to thermoelectric performance, offering clear guidance for the design of high‐efficiency nano‐thermoelectric materials.

## 2 Results and Discussion

**Figure 1:**
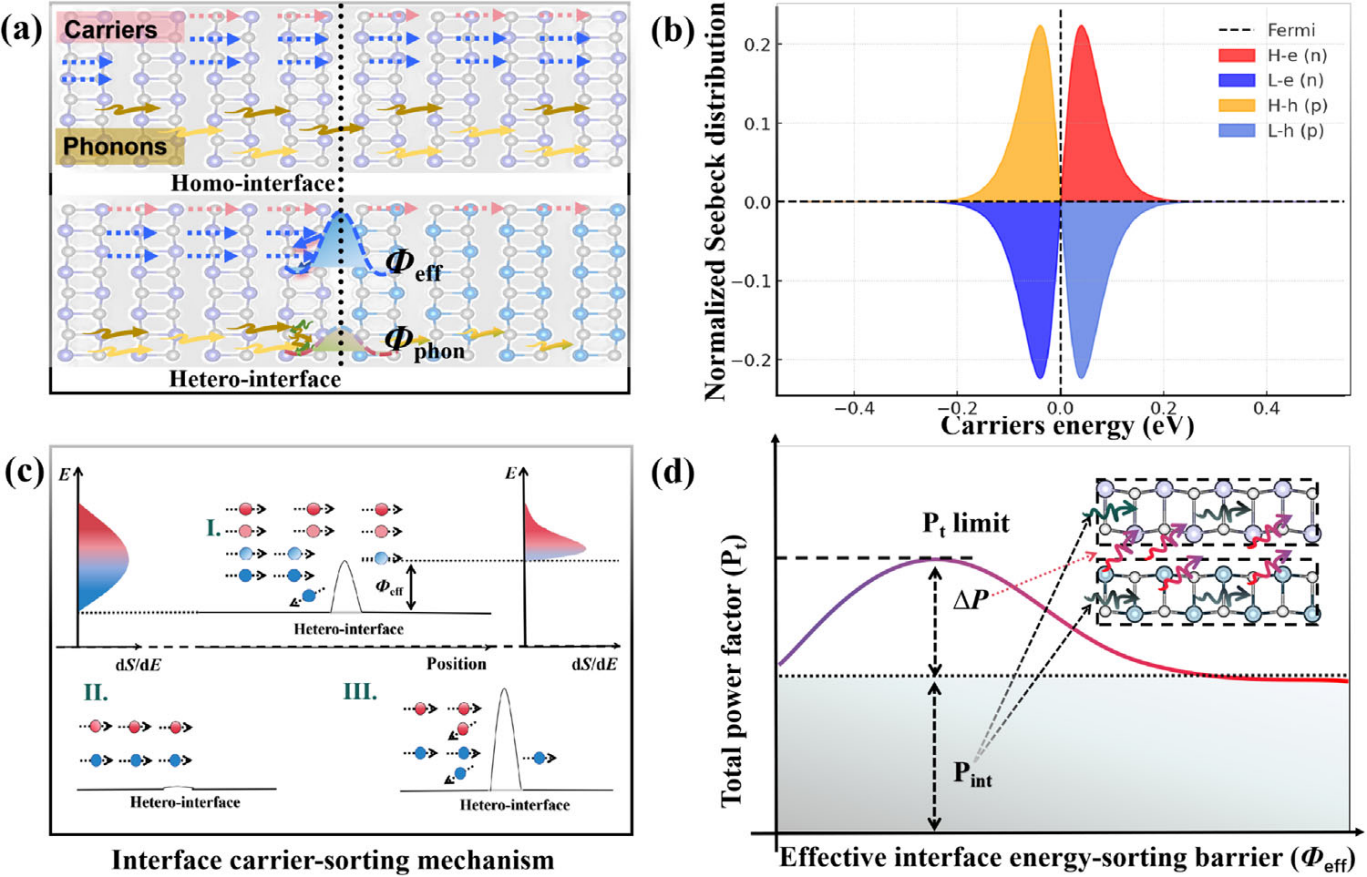
a) Carrier and phonon transport behavior through the homo‐interface and hetero‐interface. *Φ*_eff_ and *Φ*_phon_ represent the effective interfacial carrier‐sorting potential and phonon barrier, respectively. b) Relationship between carrier energy and the normalized Seebeck distribution in thermoelectric materials. Carriers with different energy levels contribute differently to the Seebeck distribution, with high‐energy carriers in a specific energy range making the most positive contribution. c) Schematic diagram illustrating the effect of different interface‐induced energy‐sorting potentials on interfacial carrier transport. Case I represents the optimal energy‐sorting carrier transport, achieving the highest *S*, while Cases II and III correspond to insufficient and excessive energy sorting, respectively. In the diagram, *Φ*_eff_ denotes the interface‐induced effective energy‐sorting potential at the heterointerface. (d) Total power factor (*P'*) as a function of *Φ*_eff_ under ideal conditions. *P_int_* is the intrinsic value without a heterointerface; Δ*P* is the interface‐induced contribution. *P'* = *P_int_* + Δ*P*.

Although heterointerfaces have been widely used to enhance thermoelectric performance, their experimental outcomes remain highly inconsistent. Theoretically, interfaces are expected to reduce *κ*_1_ through enhanced phonon scattering, but numerous studies have reported limited or even negative effects on the *P* ^[^ ^6^, ^7^, ^11^ ^]^ Such discrepancies persist even in atomically flat van der Waals (vdW) heterostructures, suggesting that phonon scattering alone cannot fully explain the observed behavior. The reduction in *κ*_1_ is typically attributed to interfacial phonon barriers arising from mismatches in phonon dispersion, vibrational modes, and lattice constants (**Figure** **1**a). However, these properties are largely intrinsic and difficult to tune. Therefore, this study shifts focus to interfacial electronic transport, which may play a critical role in determining *P* and offers a more tunable pathway for optimization. Here, we introduce carrier sorting as a generalized description of interface‐mediated modulation of carrier transport. While this concept shares a physical foundation with classical energy filtering,^[^ ^12^ ^]^ it adopts a broader perspective that accounts for partial quantum tunneling and interfacial potential smoothing, both of which are relevant in realistic heterostructures. To model this behavior, we define an effective energy‐sorting potential *Φ*_eff_ (Figure 1a), which governs the probability of carriers crossing the interface based on their energy. Unlike rigid energy barriers that completely block low‐energy carriers, *Φ*_eff_ acts as a soft barrier, reducing transmission without fully suppressing it. This allows selective enhancement of high‐energy carrier contributions, leading to an improved *S* while retaining relatively high *σ*. The *S* plays a crucial role in thermoelectric materials, characterizing the voltage response to a temperature gradient. The standard definition of the *S* is given by:^[^ ^13^ ^]^

(1) $$S=-\frac{1}{e\sigma }\int \left(\varepsilon -\mu \right)\sigma \left(\varepsilon \right)\left[-\frac{\partial f^{0}\left(\varepsilon \right)}{\partial \varepsilon }\right]d\varepsilon .$$

Here, *ɛ* represents the carrier energy, *µ* is the chemical potential, σ(*ɛ*) is the differential conductivity, and *f* ^0^(*ɛ*) is the Fermi‐Dirac distribution function. To simplify the notation, we introduce the conductivity‐weighted average energy deviation relative to the chemical potential:

(2) $$\langle \varepsilon -\mu \rangle =\frac{\int \left(\varepsilon -\mu \right)\sigma \left(\varepsilon \right)\left[-\frac{\partial f^{0}\left(\varepsilon \right)}{\partial \varepsilon }\right]d\varepsilon }{\int \sigma \left(\varepsilon \right)\left[-\frac{\partial f^{0}\left(\varepsilon \right)}{\partial \varepsilon }\right]d\varepsilon }$$

Rewriting the *S* in terms of ⟨*ɛ*−*µ*⟩, we obtain:

(3) $$S=-\frac{\langle \varepsilon -\mu \rangle }{e}.$$

However, this expression lacks an explicit temperature dependence. To account for the thermal broadening effects in electronic transport, we analyze the derivative of the Fermi‐Dirac distribution function:

(4) $$-\frac{\partial f^{0}}{\partial \varepsilon }=\frac{e^{\left(\varepsilon -\mu \right)/k_{B}T}}{{\left(e^{\left(\varepsilon -\mu \right)/k_{B}T}+1\right)}^{2}}\cdot \frac{1}{k_{B}T}$$

This expression contains a 1/*T* term, which directly influences the temperature dependence of the *S*. Thus, for dimensional consistency and correct physical interpretation, the final expression for the *S* must include a temperature factor in the denominator:

(5) $$S=-\frac{\langle \varepsilon -\mu \rangle }{eT}.$$

From Equation (5), it is evident that *S* is strongly influenced by the energy‐dependent transport properties of charge carriers relative to the chemical potential *µ*. As illustrated in Figure 1b, carriers with energies close to *µ* (low‐energy carriers) contribute minimally or even negatively to *S* (blue/light blue‐shaded region), as their transport dynamics do not significantly enhance the thermoelectric voltage. In contrast, high‐energy carriers (*ɛ* > *µ*) contribute positively to *S* (red/orange‐shaded region) due to their thermodynamically favorable transport characteristics. Thus, to enhance *S*, a *Φ*_eff_ can be introduced to selectively suppress the contribution of most low‐energy carriers, thereby shifting the charge transport distribution toward higher‐energy carriers. However, while this approach increases *S*, it may also reduce the overall carrier concentration and change carrier mobility (*µ*_e/h_), thereby decreasing the *σ*. Since the power factor is defined as *P* = *S* ^2^ *σ*, careful optimization of interfacial energy modulation is essential to balance the trade‐off between enhancing *S* and maintaining sufficient *σ* for improving *P* in thermoelectric materials. Accordingly, we obtain:

(6) $$P=\sigma \frac{{\langle \varepsilon -\mu \rangle }^{2}}{e^{2}T^{2}}.$$

As shown schematically in Figure 1a, introducing a heterointerface can create a *Φ*_eff_. Carriers must acquire energy to overcome *Φ*_eff_, shifting their accessible energy range from *ɛ* ≥ *µ* to *ɛ* ≥ *µ* + *Φ*_eff_. Mathematically, this affects both the average transport energy of the carriers and the *σ*, which is reflected in the integrals evaluated from *ɛ* = *µ* + *Φ*_eff_ to ∞. By substituting these energy limits into Equation (2), we obtain the modified power factor *P*‘as follows:

(7) $$P^{\prime }=\sigma^{\prime }\frac{{\langle \varepsilon -\mu \rangle }_{\Phi }^{2}}{e^{2}T^{2}}$$

where

(8) $${\langle \varepsilon -\mu \rangle }_{\Phi }=\frac{\int_{\mu +\Phi_{\mathrm{eff}}}^{\infty }\left(\varepsilon -\mu \right)\sigma \left(\varepsilon \right)\left[-\frac{\partial f^{0}\left(\varepsilon \right)}{\partial \varepsilon }\right]d\varepsilon }{\int_{\mu +\Phi_{\mathrm{eff}}}^{\infty }\sigma \left(\varepsilon \right)\left[-\frac{\partial f^{0}\left(\varepsilon \right)}{\partial \varepsilon }\right]d\varepsilon }$$

and

(9) $$\sigma^{\prime }=\int_{\mu +\Phi_{\mathrm{eff}}}^{\infty }\sigma \left(\varepsilon \right)\left[-\frac{\partial f^{0}\left(\varepsilon \right)}{\partial \varepsilon }\right]d\varepsilon .$$

Physically, suppressing the contribution of most low‐energy carriers increases the average transport energy, thereby improving the *S*. However, it also reduces the overall carrier concentration, which lowers the *σ*'.Consequently, the resulting *P*' critically depends on balancing these opposing effects. As shown in Figure 1c, a smaller *Φ*_eff_ can increase *S* enough to outweigh the drop in *σ*′, improving *P*′. However, an excessively large *Φ*_eff_ significantly reduces carrier density, leading to a decline in *P*′. This explains why introducing heterointerfaces does not always result in an enhancement of the power factor in experiments. Thus, a well‐designed interface with an optimal *Φ*_eff_ ​can effectively preserve the intrinsic *P*_int_ of the material while regulating the carrier energy distribution. This optimization maximizes the enhancement of the interface‐induced extrinsic Δ*P*, bringing it closer to the theoretical total power factor (*P*′) limit of the material (Figure 1d).

The primary physical mechanism by which heterointerfaces enhance thermoelectric performance lies in their ability to modulate charge carrier transport and energy distribution. This modulation influences extrinsic thermoelectric parameters, including the extrinsic Seebeck coefficient (Δ*S*), electrical conductivity (Δ*σ*), and power factor (Δ*P*), which collectively reflect the interface's impact on microscopic transport mechanisms. While previous experiments have qualitatively demonstrated the role of interfacial transport effects in improving thermoelectric *ZT*, the underlying mechanisms and the precise dependence of extrinsic thermoelectric parameters on effective *Φ*_eff_ remain unclear.^[^ ^11^, ^14^ ^]^ To systematically investigate carrier‐sorting transport at heterointerfaces, we propose a superlattice model composed of alternating SnSe and GeSe layers (Figure S1, Supporting Information). To systematically investigate carrier‐sorting transport at heterointerfaces, we propose a superlattice model composed of alternating SnSe and GeSe layers (Figure S1, Supporting Information). This material platform is particularly well‐suited for such modeling due to its advantageous structural and electronic characteristics. Compared to conventional heterostructures such as Bi₂Te₃/Sb₂Te₃ and PbTe/PbSe, the SnSe/GeSe superlattice offers several key advantages. These include ultralow *κ*_l_, multivalley band structures that enhance *S*, and clean vdW interfaces that minimize defect‐induced scattering. GeSe provides excellent lattice matching with SnSe and a small work function difference, enabling coherent interface formation. SnSe, known for its exceptional thermoelectric performance, exhibits a bulk figure of merit up to ZT ≈ 2.6–2.8,^[^ ^15^ ^]^ providing a strong foundation for high‐efficiency energy conversion. Both SnSe and GeSe are composed of non‐toxic, earth‐abundant elements and can be synthesized via low‐temperature solution‐phase^[^ ^16^ ^]^ or CVD methods,^[^ ^17^ ^]^ supporting scalable and environmentally friendly fabrication. By using the intrinsic transport characteristics of each constituent material's pristine supercell as a baseline called “Δ‐method”, we extract the extrinsic Δ*S*, Δ*σ*, and Δ*P* induced by carrier‐sorting transport at the interfaces. This approach reduces the influence of bulk material properties, ensuring that the *Φ*_eff_ predominantly governs cross‐interface carrier transport and enhances the model's universality.

**Figure 2:**
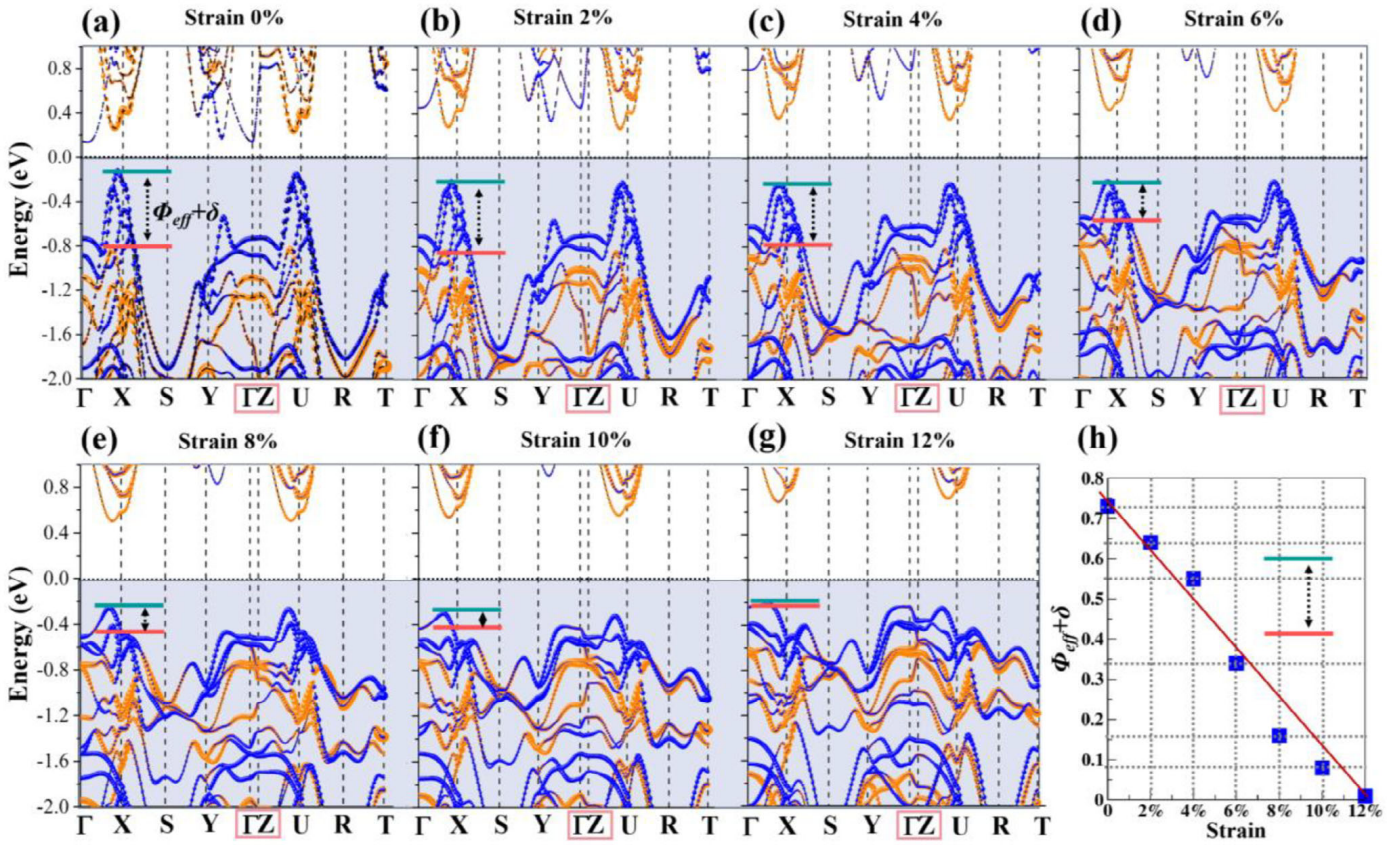
a–g) The evolutional control of the valence band offsets (Δ*E, Φ*_eff_+*δ*) in a hetero‐structural model is achieved through the continuous application of biaxial strain. The red and green short bars represent the valence band edges of the two materials SnSe and GeSe, respectively. h) The (*Φ*_eff_+*δ*) shows a linear relationship with the biaxial strain adjustment.

To facilitate the quantitative description of the relationship between the interfacial energy‐sorting effect and interfacial electronic properties, the effective potential *Φ*_eff_ is approximated as Δ*E*−*δ*, where Δ*E* corresponds to the valence band offset between SnSe and GeSe. This offset has traditionally been interpreted as a rigid barrier and is commonly associated with classical carrier filtering mechanisms.^[^ ^12^ ^]^ The correction term *δ* accounts for the softening of the barrier due to partial quantum tunneling and interfacial potential smoothing. Interestingly, our band structure calculations reveal that by applying biaxial strain, Δ*E*(*Φ*_eff_+*δ*) can be tuned from 0 to 0.73 eV (**Figure** **2**). It is worth noting that this strain also affects band dispersion. To eliminate the influence of these changes on transport properties, we constructed both SnSe and GeSe supercells matching the dimensions of the superlattice and applied the same biaxial strain to both supercells. These serve as calibrations to isolate the effects of strain‐induced band dispersion in the SnSe/GeSe superlattice. We focus on the electronic structure along the *Γ*‐*Z* path because the impact of the heterointerface on thermoelectric performance is primarily reflected in the carrier transport across the junction. The electronic structure along this path reveals key mechanisms of interlayer thermoelectric transport in the heterostructure, particularly the effects of vdW tunneling barriers and interface‐induced energy‐sorting effect on carrier transport. Along the *Γ* *‐Z* path, we observed relatively flat electronic bands, primarily due to weak interlayer coupling across the vdW gaps and effective *Φ*_eff_ at the heterointerfaces, which limit carrier dispersion. As strain increases, these bands become even flatter, exhibiting a progressive reduction in band dispersion. To further investigate the underlying mechanism for the reduced band dispersion along the *Γ*‐*Z* path under increasing strain, we summarized the interlayer distances of the heterostructures under different strain conditions in Table S1 (Supporting Information). The results reveal minimal changes in interlayer distance, suggesting that the reduction in band dispersion is primarily driven by strain‐induced modifications in *Φ*_eff_+*δ*.

**Figure 3:**
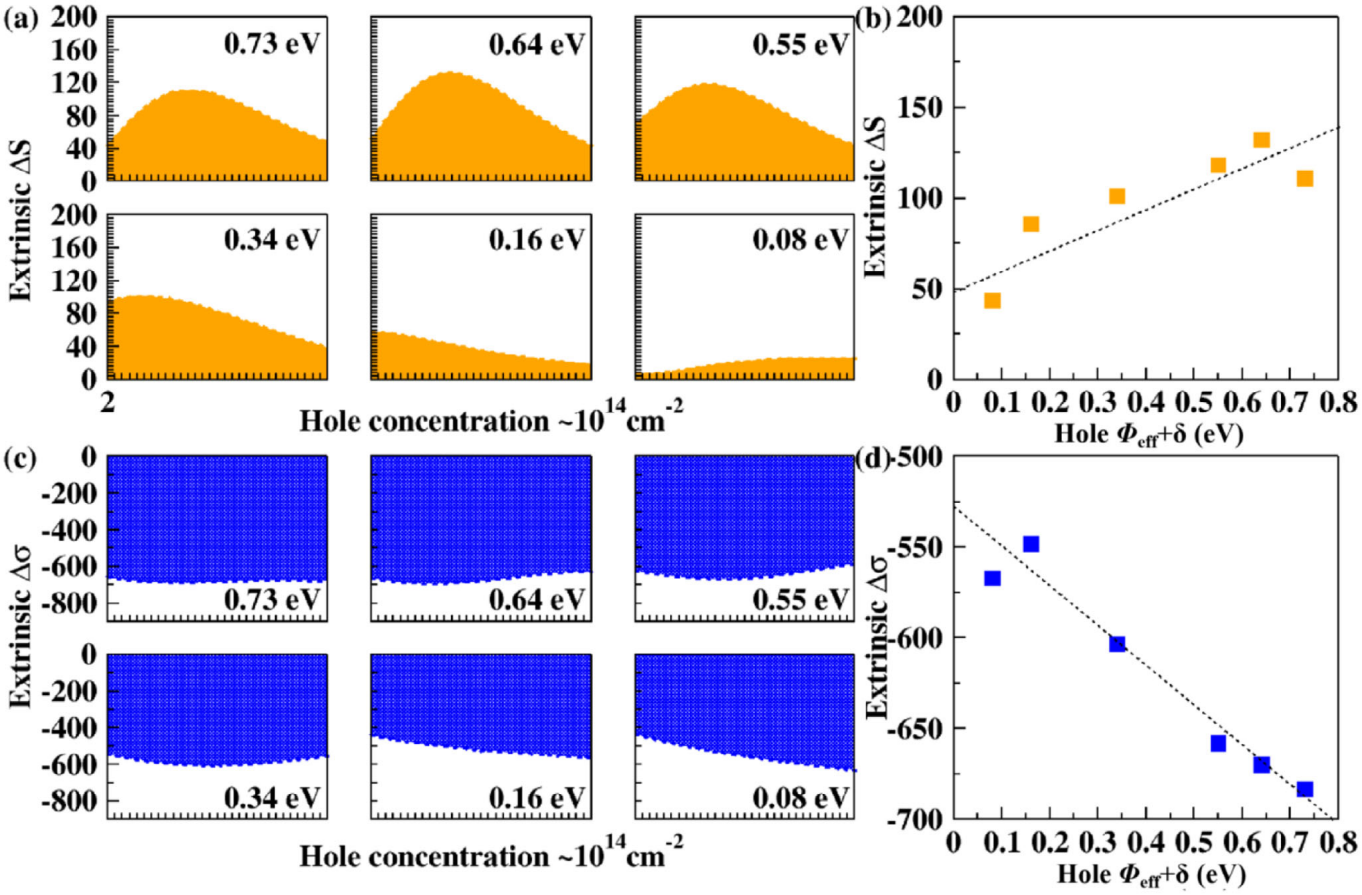
a) Influence of different (Δ*E*, *Φ*_eff_+*δ*) on carrier‐sorting transport, leading to variations in the extrinsic Δ*S* at 800 K. b) With an increase in interface potential at a specific hole concentration, the Δ*S* peak is correspondingly altered. c) At 800 K, variations in (*Φ*_eff_+*δ*) affect carrier transport, leading to different reductions in extrinsic Δ*σ*. d) The Δ*σ* corresponding to the ∆*S* peak shown in Figure 3b varies with the change in (*Φ*_eff_+*δ*). Here, the unit of ∆*S* is µV/K, and the unit of ∆*σ* is 10^2^ Ω⁻¹·m⁻¹.

Building on the previously computed electronic structures of the SnSe/GeSe superlattice under a series of different (*Φ*_eff_+*δ*) values (Figure 2), we further investigated its thermoelectric transport properties, including the *S*, *σ*, and *P* (Figures S2–S4, Supporting Information). To isolate interfacial effects from bulk band dispersion, we also computed the electronic band structures (Figures S5 and S6, Supporting Information) and corresponding thermoelectric parameters for 1 × 1 × 4 SnSe and GeSe supercells along the *z*‐axis under identical strain conditions. To establish a reference baseline, we averaged the thermoelectric parameters of these supercells and compared the transport properties of the SnSe/GeSe superlattice against this baseline. This approach effectively isolates the effects of the tunneling barriers from vdW gap and strain‐induced lattice distortions on carrier transport. Given that bulk SnSe achieves its optimal thermoelectric performance at ≈800K, we set the temperature at 800K.^[^ ^15b^ ^]^ Through this comparative analysis, we extracted Δ*S* and Δ*σ* induced by interfacial energy‐sorting effect at 2D heterointerfaces under different (*Φ*_eff_+*δ*), as shown in **Figure** **3**. Figure 3a illustrates that Δ*S* increases with (*Φ*_eff_+*δ*), primarily due to the stronger selective control of carrier transport at higher *Φ*_eff_, which suppresses the contribution of partial low‐energy carriers and enhances the overall *S*. In non‐degenerate semiconductors, where carrier transport follows Boltzmann statistics, the shift in mean transport energy leads to a nearly linear dependence of the *S* on *Φ*_eff_, given by

(10) $$\Delta S\approx \left|\frac{1}{eT}\cdot \Phi_{\mathrm{eff}}\right|\approx \left|\frac{1}{eT}\cdot \left(\Delta E-\delta \right)\right|.$$

However, in degenerate semiconductors, where charge carriers obey Fermi‐Dirac statistics, the enhancement of ∣*S*∣ is reduced due to the finite occupation of electronic states near the Fermi level. In this case, the change in *S* is modified by a Fermi integral correction factor, yielding

(11) $$\Delta S\approx \left|\frac{1}{eT}\cdot \frac{F_{1}\left(\eta \right)}{F_{0}\left(\eta \right)}\cdot \Phi_{\mathrm{eff}}\right|\approx \left|\frac{1}{eT}\cdot \frac{F_{1}\left(\eta \right)}{F_{0}\left(\eta \right)}\cdot \left(\Delta E-\delta \right)\right|$$

where *F_n_* (*η*) are Fermi integrals, and *η* = *µ*/*k*_B_ *T* is the reduced chemical potential. Since *F*_1_(*η*)/*F*_0_(*η*)<1, the impact of ∆*E* on |*S*∣ is weaker in degenerate systems compared to non‐degenerate cases. We found that the theoretically derived dependence of Δ*S* on ∆*E* (*Φ*_eff_ + *δ*) is in good agreement with the computationally obtained trend, as shown in Figure 3b. As illustrated in Figure 3c, the exclusion of partial lower‐energy carriers reduces *σ* primarily due to a decrease in carrier mobility. In degenerate semiconductors, where the Fermi level is well within the conduction/valence bands, the total carrier concentration remains relatively stable despite moderate variations in *Φ*_eff_. Therefore, *Φ*_eff_ primarily affects carrier mobility. Specifically, enhanced interfacial scattering further reduces mobility, thereby weakening *σ*. As shown in Figure 3d, within the studied range, *σ* decreases approximately linearly with increasing *Φ*_eff_. Meanwhile, this potential selectively suppresses partial low‐energy carriers, effectively shifting the average transport energy toward higher values, which results in an approximately linear increase in the Seebeck coefficient (|*S*|). Since |*S*| increases nearly linearly while *σ* decreases similarly, the power factor *P* = *S* ^2^ *σ* exhibits an overall quadratic dependence on the *Φ*_eff_. This suggests the existence of an optimal band offset (Δ*E*_opt_ ≈ *Φ*_opt_ + *δ*), which balances the trade‐off between enhancing |*S*| and reducing *σ*, thereby maximizing thermoelectric performance.

**Figure 4:**
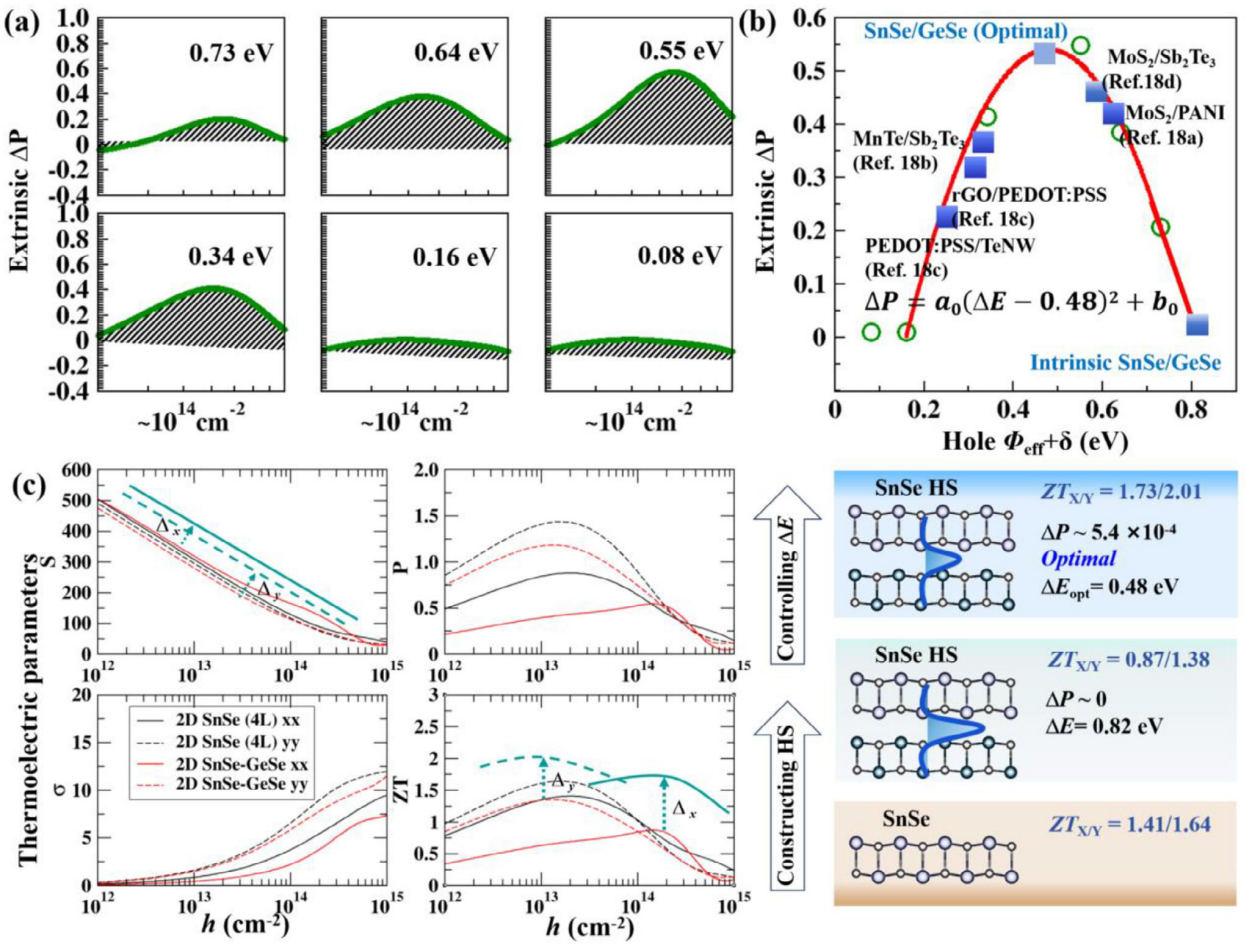
a) The variation of the extrinsic power factor (Δ*P*) with increasing (*Φ*_eff_ + *δ*). b) The volcano diagram of Δ*P* to Δ*E* (*Φ*_eff_ + *δ*). The green hollow circle represents power factor gains at specific Δ*E*, while the red solid line indicates the fitted Δ*P* ‐ Δ*E* relationship. Blue solid squares represent experimental data, indicating the corresponding Δ*E* to the *p*‐type Δ*P*‐optimization achieved experimentally.^[^ ^18^ ^]^ c) Comparison of the transport properties in the *x* and *y* directions for four‐layer pure SnSe and SnSe/GeSe heterostructures. The red solid and dashed lines represent the thermoelectric transport parameters and *ZT* in the *x* and *y* directions at a Δ*E* of 0.82 eV. The teal solid and dashed lines indicate the thermoelectric parameters and maximum achievable *ZT* in the *x* and *y* directions for the SnSe/GeSe heterostructure at the optimal Δ*E* of 0.48 eV. Here, Δ*S* is measured in µV/K, ∆*σ* is in 10^2^ Ω^−1^m^−1^, and Δ*P* is in 10^−3^ Wm^−1^K^−2^, respectively.

To determine the optimal (*Φ*_eff_ + *δ*) corresponding to the peak enhancement of the Δ*P*, we systematically investigated the variation of Δ*P* within an (*Φ*_eff_ + *δ*) of 0 to 0.73 eV. As shown in **Figure** **4**a, for a given carrier concentration, Δ*P* initially increases with increasing *Φ*_eff_ before reaching a peak and subsequently decreasing within a specific range of carrier concentrations. Additionally, we observed that as (*Φ*_eff_ + *δ*) increases, both the average extrinsic Δ*P* and its peak value exhibit a non‐monotonic trend, first rising to a maximum and then gradually declining. Through numerical fitting, we found that the relationship between the extrinsic Δ*P* and the (*Φ*_eff_ + *δ*) follows an approximate quadratic dependence: Δ*P* = −*a*(*Φ*_eff_ + *δ* − 0.48) + *b* = −*a*(Δ*E* − 0.48) + *b*, where *a* = 5.27 × 10^−3^ Wm^−1^K^−2^eV^−2^, *b* = 5.40 × 10^−4^ Wm^−1^K^−2^. The quadratic dependence suggests that moderate *Φ*_eff_ enhances charge transport efficiency, while excessively large *Φ*_eff_ hinders carrier transmission, thereby reducing Δ*P*. It is worth noting that the optimal value of *Φ*_eff ​_ may vary between material systems due to differences in the softening correction *δ*, which reflects interface‐specific effects. Nevertheless, the qualitative dependence of Δ*P* on the band offset Δ*E* remains consistent across different systems. For relatively clean interfaces where *δ* is typically less than 100 meV, Δ*E* serves as a close approximation to the *Φ*_eff_, making it a practically useful and computationally accessible design parameter. In contrast, in structurally complex or disordered interfaces, larger *δ* values can limit the accuracy of using Δ*E* as a sole descriptor. As shown in Figure 4b, the extrinsic Δ*P* remains relatively high within an Δ*E* range of 0.30 to 0.70 eV, indicating that within this window, energy‐sorting carrier transport is optimized for thermoelectric enhancement. Notably, when Δ*E* is ≈0.48 eV, the peak power factor reaches 0.54 × 10^−3^ Wm^−1^K^−2^. To validate the predictive capability of our model, we first examined previously reported experimental data from various heterostructures.^[^ ^18^ ^]^ We then conducted a detailed case study on a representative system, Sb₂Te₃/MoS₂, which provides available experimental measurements for comparison.^[^ ^18d^ ^]^ Using a computational interface model (Figure S7a, Supporting Information), we simulated the interfacial transport behavior under similar conditions. Although the model size is smaller than that of experimental samples due to computational limitations, it sufficiently captures the key carrier transport across the interface. As shown in Figure S7b (Supporting Information), when the valence Δ*E* is ≈0.6 eV, the predicted Δ*S* closely matches the experimental values. The calculated trends of Δ*σ* and Δ*P* also show good agreement in the low‐temperature regime. At intermediate temperatures, minor deviations in Δ*σ* are observed, which we attribute to differences in carrier concentration behavior. Specifically, the carrier concentration in Sb₂Te₃/MoS₂ remains nearly constant with temperature, while pure Sb₂Te₃ exhibits a strong temperature dependence. To more accurately reflect the experimental scenario, we adopted a fixed carrier concentration in our simulations. Furthermore, by tuning Δ*E* via strain, we reproduced thermoelectric behaviors that are consistent with those observed in the SnSe/GeSe system (Figure S8, Supporting Information). This cross‐system consistency strongly supports the robustness and general applicability of our theoretical framework.

To consolidate these findings, we present a general theoretical framework for understanding and designing interface‐driven thermoelectric systems, with three key innovations. First, we develop a general physical model based on the effective *Φ*_eff_, which moves beyond the conventional assumption of static band alignment by treating interfacial modulation as a continuous, tunable parameter. Second, we introduce the Δ‐method to quantitatively separate interface‐induced contributions to the *S*, *σ*, and *P*. This reveals a nonlinear volcano‐shaped dependence of power factor on *Φ*_eff_ and identifies, for the first time, a feasible optimization window for band offsets, offering practical guidance for interface engineering. Third, our theory model explicitly accounts for vertical heat flow in multilayered structures, capturing its additional contribution to thermoelectric performance. This refinement improves the relevance of theoretical predictions to real devices and helps bridge the gap between simulation and experimental observation.

Based on our predicted Δ*P* ‐ Δ*E* relationship, we next aim to further explore the maximum achievable *ZT* in 2D SnSe/GeSe by optimizing interfacial contributions. Compared to bilayer structures, the four‐layer SnSe/GeSe heterostructure not only has a more suitable thickness, making it easier to synthesize, but also demonstrates greater potential for practical applications. Therefore, we employ a four‐layer SnSe‐based heterostructure, created by vertically stacking bilayers of SnSe and GeSe, as a model to investigate the potential for enhancing the thermoelectric performance of 2D materials through optimized interfacial energy modulation. Our electronic structure calculations (Figure S9, Supporting Information), performed at the HSE06 hybrid functional^[^ ^19^ ^]^ level, show that interlayer interactions at the intrinsic heterojunction induce slight changes in band dispersion and degeneracy. More importantly, the *Φ*_eff_ of this four‐layer intrinsic SnSe/GeSe heterostructure is 0.82 eV. According to our predicted Δ*P* ‐ Δ*E* relationship, the energy‐dependent regulation of carrier transport in this intrinsic heterostructure is excessively strong, leading to a reduction in both the average and maximum power factors (Figure 4c). This further emphasizes the importance of optimizing the *Φ*_eff_. If the interfacial energy modulation in the four‐layer SnSe/GeSe heterostructure can be optimized to a Δ*E* (*Φ*_eff_ + *δ*) of ≈0.48 eV, the *S* along the *x*‐ and *y*‐directions would increase significantly. By fully compensating for the reduction in *σ* caused by the heterostructure, the *P* of 2D SnSe could be improved by 25%, raising its *ZT* value (along the x/y‐directions) from 1.47/1.64 to 1.73/2.01 (Figure 4d). By compensating for the reduction in electrical conductivity induced by the heterostructure, the power factor of 2D SnSe can be enhanced by ≈25%, resulting in *ZT* values increasing from 1.47/1.64 to 1.73/2.01 along the x/y directions (Figure 4d). This positions the SnSe/GeSe system as a highly competitive candidate among 2D thermoelectric materials. Nevertheless, these predicted values are based on idealized structural models, and their realization in practical devices may be limited by non‐ideal effects. For example, although interface phonon scattering contributes to lowering lattice thermal conductivity, interfacial roughness or structural discontinuities may also enhance carrier scattering, thereby reducing mobility and suppressing *P* gains. Moreover, fabrication‐related imperfections, such as thickness fluctuations or interfacial defects, could alter the intended band alignment and introduce parasitic resistance. To address these challenges, several practical strategies can be considered, including precise control of growth parameters, atomic‐scale thickness engineering using techniques such as atomic layer deposition, and tunable modulation of *Φ*_eff_ through composition adjustment and external electric fields. These findings allow for rapid assessment of specific heterostructures in enhancing thermoelectric performance, significantly reducing experimental costs. Additionally, this study provides strong theoretical guidance for surpassing the conventional *ZT* limit through the interface‐driven carrier sorting mechanisms.

**Figure 5:**
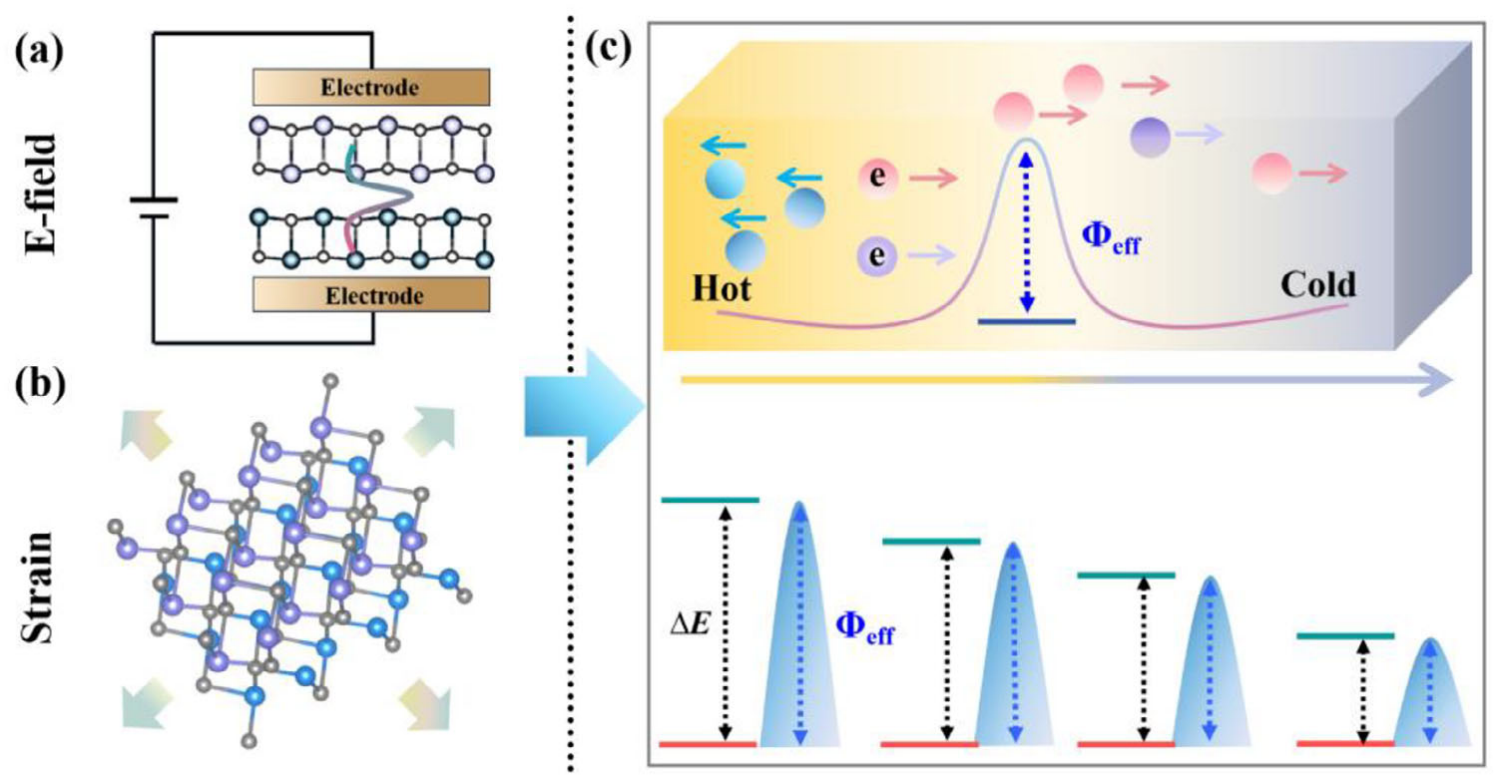
a–c) Control and measurement of effective energy‐sorting potential (*Φ*_eff_). In semiconductor‐based heterostructures, *Φ*_eff_ + *δ* at the heterointerface can be characterized by the band offset, ∆*E*. This ∆*E* (*Φ*_eff_ + *δ*), is a measurable quantity that can be continuously tuned in experiments by applying external electric fields (a) or strain (b). The red and green solid lines represent the valence or conduction band edge positions of the two materials forming a heterostructure, respectively.

From an experimental perspective, the proposed Δ*E*, which is closely related to the effective *Φ*_eff_, can be directly measured using techniques such as X‐ray photoelectron spectroscopy (XPS) combined with ultraviolet photoelectron spectroscopy (UPS)^[^ ^20^ ^]^ or angle‐resolved photoelectron spectroscopy (ARPES).^[^ ^21^ ^]^ To validate the reliability of our density functional theory (DFT) calculations for Δ*E*, we systematically compiled and compared experimental measurements (e.g., from XPS, UPS, and ARPES) with DFT results across a range of similar layered heterostructures, as summarized in Table S2 ^[^ ^22^ ^]^ (Supporting Information). The comparison shows good agreement, with deviations typically within 0.1 eV, confirming both the accuracy and experimental relevance of the calculated band offsets. By applying external electric fields (**Figure** **5**a) or strain engineering (Figure 5b), researchers can precisely tune Δ*E*, resulting in shifts of the valence band maximum and the conduction band minimum and modifications of the Δ*E* (Figure 5c).^[^ ^23^ ^]^ These approaches have been successfully demonstrated in devices like field‐effect transistors (FETs), confirming both the feasibility and the broad applicability of this method for optimizing electronic and thermoelectric properties in heterostructures.

Despite its promising thermoelectric properties, the SnSe/GeSe system still faces several key challenges for large‐scale device integration. First, achieving atomically smooth and chemically clean interfaces over large areas is difficult. Elemental interdiffusion, lattice mismatch, and surface roughness can introduce defects and inhomogeneities, reducing interfacial transport stability. Second, while techniques such as molecular beam epitaxy (MBE)^[^ ^24^ ^]^ and mechanical transfer offer high precision, they are low in efficiency and not suitable for industrial‐scale production. To address these challenges, several promising approaches have been developed. Low‐temperature solution synthesis (<300 °C)^[^ ^16^ ^]^ has been successfully applied to SnSe@GeSe nanosheets, offering good scalability and cost advantages. CVD)^[^ ^17^ ^]^ enables precise control over thickness and crystallographic orientation in IV–VI materials, which may help improve lattice matching and interface quality in SnSe/GeSe. In addition, centimeter‐scale deterministic transfer and lamination techniques have been widely demonstrated in vdW heterostructures and can also be applied to achieve consistent interfaces in the SnSe/GeSe system.

## 3 Conclusion

In summary, this study establishes a quantitative framework for investigating the role of effective *Φ*_eff_ in enhancing carrier‐sorting transport mechanisms and optimizing thermoelectric performance in heterostructures. Using a SnSe/GeSe superlattice stacked along the *z*‐direction, we quantitatively extracted the extrinsic thermoelectric parameters induced by the *Φ*_eff_ by comparing them with the baseline properties of SnSe and GeSe supercells. Through biaxial strain engineering, we demonstrated that the p‐type *Φ*_eff_ at the SnSe/GeSe interface, can be precisely tuned. First‐principles calculations combined with Boltzmann transport theory reveal that the extrinsic Δ*S*, Δ*σ* and Δ*P* evolve systematically with Δ*E* (*Φ*_eff_ + *δ*). Further analysis identifies an optimal Δ*E*_opt_ (*Φ*_opt_ + *δ*) of ≈0.48 eV, at which Δ*P* reaches its peak, aligning well with previous experimental results and validating our theoretical model. We predict that a four‐layer SnSe/GeSe heterostructure with an optimized Δ*E*_opt_ can achieve a *ZT* value of 2.01. Beyond this specific system, the presented framework offers generalizable insights into the design and optimization of heterostructure‐based thermoelectrics by highlighting the central role of interfacial energy‐related transport. These findings bridge the gap between atomistic modeling and device‐scale performance prediction, advancing our understanding of how interfacial phenomena can be harnessed to approach or exceed current limits in nanoscale thermoelectric systems.

## 4 Experimental Section

For the computational model, a superlattice consisting of alternating bilayers of SnSe and GeSe was constructed. Each superlattice unit includes 4 Sn atoms, 4 Ge atoms, and 8 Se atoms, with an interfacial distance of ≈2.26 nm between adjacent SnSe/GeSe layers (Figure S1, Supporting Information). At this interface spacing, the effect of potential density on carrier transport can be disregarded, and the influence of the effective interfacial energy‐sorting potential (*Φ*_eff_) is approximated as stemming from a single potential. To accurately quantify the extrinsic thermoelectric parameters induced by the *Φ*_eff_, SnSe and GeSe supercells of the same size were selected, and their averaged thermoelectric parameters were used as a reference baseline. Additionally, to estimate the maximum achievable 2D thermoelectric *ZT* under optimal *Φ*_eff_, a four‐layer SnSe/GeSe heterostructure, composed of a SnSe bilayer and a GeSe bilayer, was employed, as illustrated in Figure S9 (Supporting Information).

All first‐principles calculations were performed using the Vienna Ab initio Simulation Package (VASP),^[^ ^25^ ^]^ based on Density Functional Theory (DFT).^[^ ^26^ ^]^ Projector‐augmented wave (PAW) potentials^[^ ^27^ ^]^ and the Perdew‐Burke‐Ernzerhof (PBE) exchange‐correlation functional^[^ ^28^ ^]^ were employed, with a plane‐wave cutoff energy of 500 eV. Both lattice geometries and atomic positions were optimized until the forces converged to less than 0.01 eV Å^−1^, and the energy difference between iterations was below 1 × 10⁻⁵ eV. A vacuum layer of 20 Å was introduced to prevent interactions between adjacent layers. The DFT + D3^[^ ^29^ ^]^ approach was used to accurately model the vdW interactions between layers. As Sn, Ge, and Se are not heavy elements, spin‐orbit coupling (SOC) was not included in the calculations. In the electronic structure calculations of the superlattices and supercells, the primarily focus was placed on the band edges and normalized the effect of the bandgap on transport properties. This approach significantly reduced the influence of bandgap variations on the extrinsic thermoelectric parameters (Δ*S*, Δ*σ* and Δ*P*). Given that the PBE functional provides a reasonable balance between accuracy and computational cost, all electronic structure calculations for the superlattices and supercells were performed using PBE. However, predicting the thermoelectric performance of 2D systems requires a more precise estimation of the bandgap. Therefore, for the electronic structure and thermoelectric transport property predictions of the 2D SnSe/GeSe structures, the HSE06 hybrid functional^[^ ^19^ ^]^ was employed to improve accuracy. The plane‐wave cutoff energy was set to 500 eV, and the energy convergence criterion was tightened to 1 × 10⁻⁶ eV to ensure high precision in the calculations.

The thermoelectric parameters, namely the Seebeck coefficient and electrical conductivity normalized to a constant relaxation time, were calculated using the BoltzTrap2 code,^[^ ^30^ ^]^ based on electronic band structures derived from PBE (superlattices and supercells) or HSE06 calculations (four‐layer SnSe/GeSe and pure SnSe system). The direction‐dependent relaxation times, *τ*_i_ (i = *_x_*_,_ *_y_*_,_ *_z_*_)_, were determined by combining temperature‐ and carrier‐concentration‐dependent experimental data^[^ ^15^, ^31^ ^]^ with the theoretical calculations, yielding more reliable results than those derived solely from deformation potential theory.^[^ ^32^ ^]^

## Conflict of Interest

The authors declare no conflict of interest.

## Supporting information



Supporting Information

## Data Availability

The data that support the findings of this study are available from the corresponding author upon reasonable request.
