# Supplementary material for: Toward the Thermoelectric ZT Limit via the Quantification of Interface‐Driven Carrier Sorting
Source: Small. 2025 Jul 25;21(37):e05325. doi: 10.1002/smll.202505325 (PMC12444869; doi:10.1002/smll.202505325)
Supplement: Supplementary file 1 — Supporting Information [file SMLL-21-e05325-s001.docx]

Supporting Information

**Toward the Thermoelectric ZT Limit via the Quantification of Interface-Driven Carrier Sorting**

Xiwen Zhang, Yiming Zhao, Liang Ma, Zhensen Chen, Yunfei Chen*, Jinlan Wang*, and Lei Shen*

**Table of Contents**

1. Lattice models for studying interfacial carrier transport mechanisms.
2. Calculated Seebeck coefficients of SnSe/GeSe superlattices and SnSe/GeSe supercells along the *Γ-Z* direction under interface-engineered valence band offsets.
3. Calculated electrical conductivity of SnSe/GeSe superlattices and SnSe/GeSe supercells along the *Γ-Z* direction under interface-engineered valence band offsets.
4. Calculated power factors of SnSe/GeSe superlattices and SnSe/GeSe supercells along the *Γ-Z* direction under interface-engineered valence band offsets.
5. Electronic band structure of the 1×1×4 SnSe supercell as a reference under conditions of continuously varying strain.
6. Electronic band structure of the 1×1×4 GeSe supercell as a reference under conditions of continuously varying strain.
7. Structure models and a comparison of experimental and theoretical thermoelectric parameters of Sb₂Te₃/MoS₂ heterostructure.
8. The evolution of the additional thermoelectric parameters for Sb₂Te₃/MoS₂ heterostructures under three distinct strain-induced band offsets.
9. Lattice structures and electronic band structures of SnSe/GeSe heterostructure and SnSe four-layer.
10. Interlayer distance at heterointerfaces in SnSe/GeSe superlattices under different strains.
11. Experimental vs. DFT-calculated band offsets for representative heterostructures.


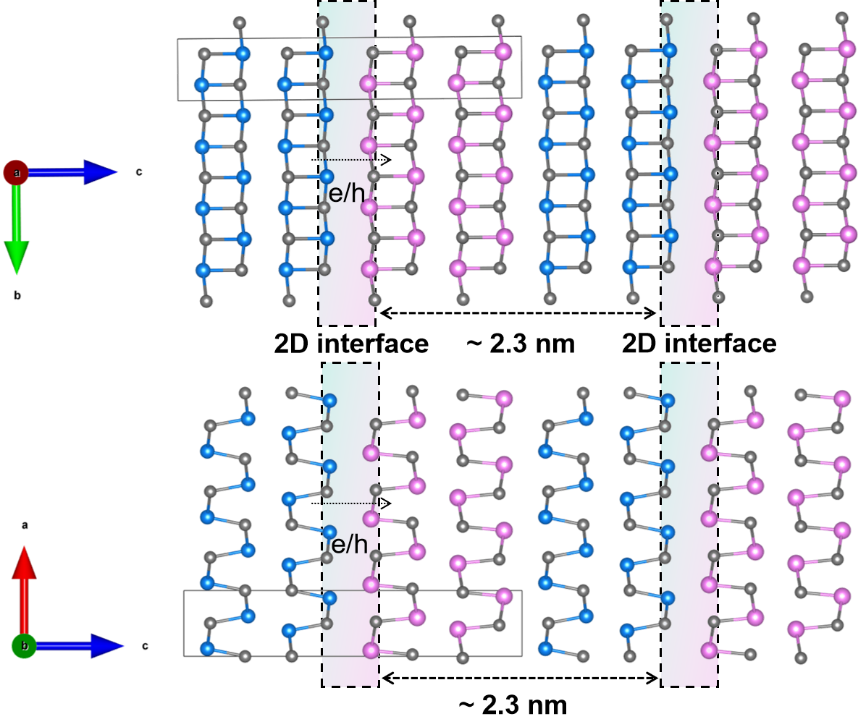


**Figure S1.** Atomic structure of the SnSe/GeSe superlattice model. The SnSe/GeSe superlattice model consists of alternating bilayers of SnSe and GeSe, with a nearest-interface spacing of approximately 2.3 nm.

**
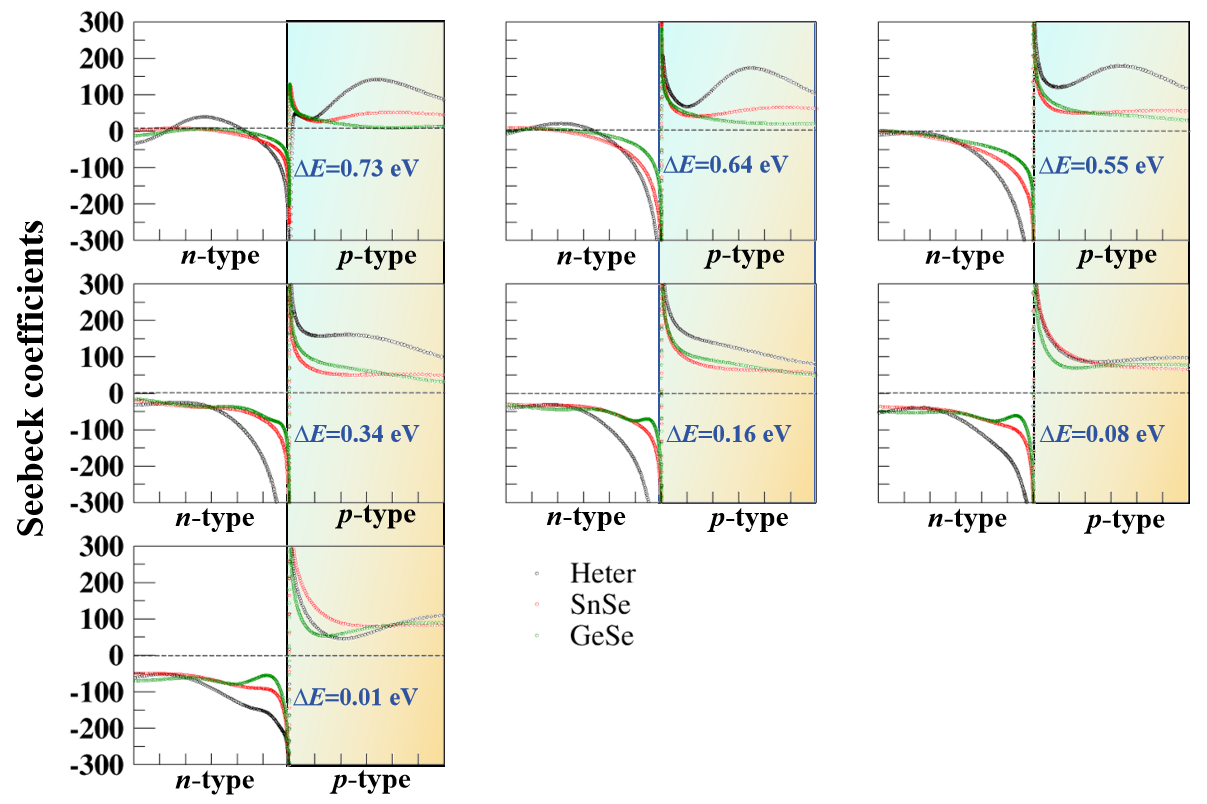
**

**Figure S2.** Calculated Seebeck coefficients along the *Γ-Z* direction. Calculated Seebeck coefficients of SnSe/GeSe superlattices and SnSe/GeSe supercells along the *Γ-Z* direction under interface-engineered valence band offsets. To minimize errors from bandgap fluctuations, the Seebeck coefficients of SnSe and GeSe supercells were determined by adjusting their bandgaps to match those of the SnSe/GeSe superlattice using a bandgap shearing method. All Seebeck coefficients are expressed in *μ*V/K.


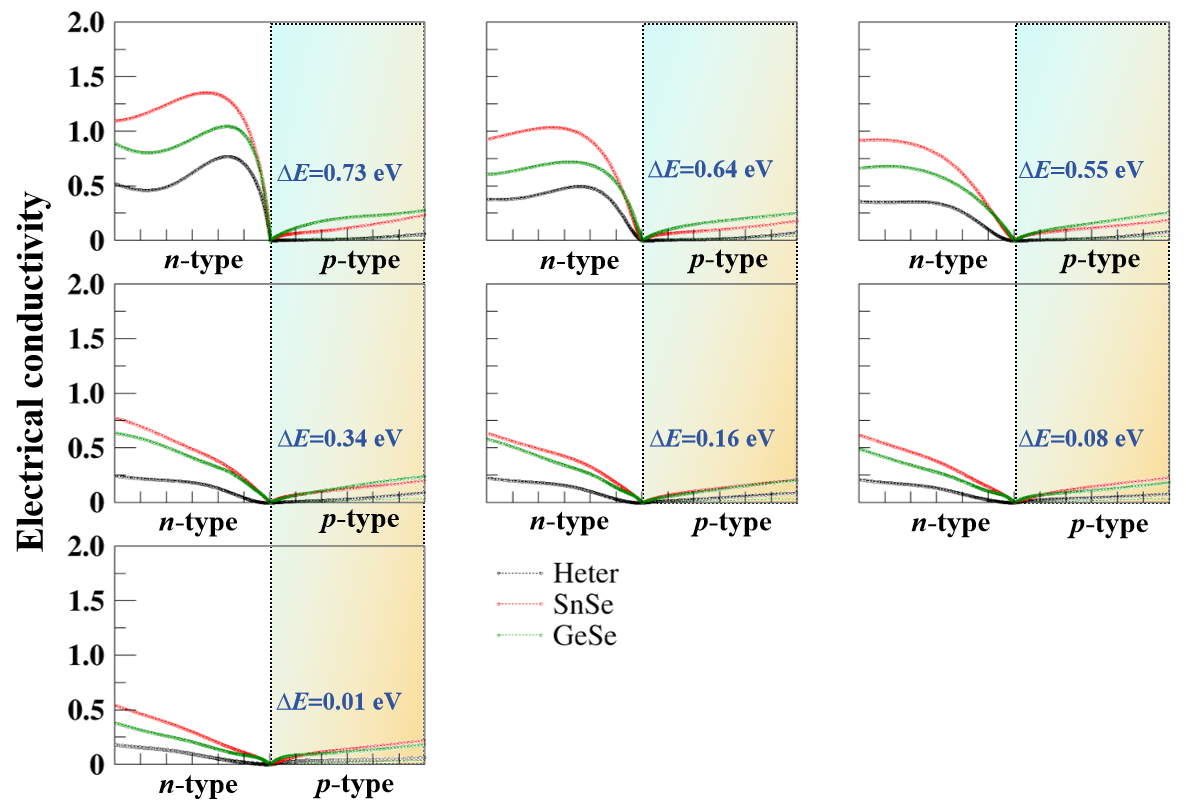


**Figure** **S3.** Calculated electrical conductivity along the *Γ-Z* direction. Calculated electrical conductivity of SnSe/GeSe superlattices and SnSe/GeSe supercells along the *Γ-Z* direction under interface-engineered valence band offsets. Electrical conductivity values are expressed in units of *τ*⁻¹10²⁰ Ω⁻¹m⁻¹.


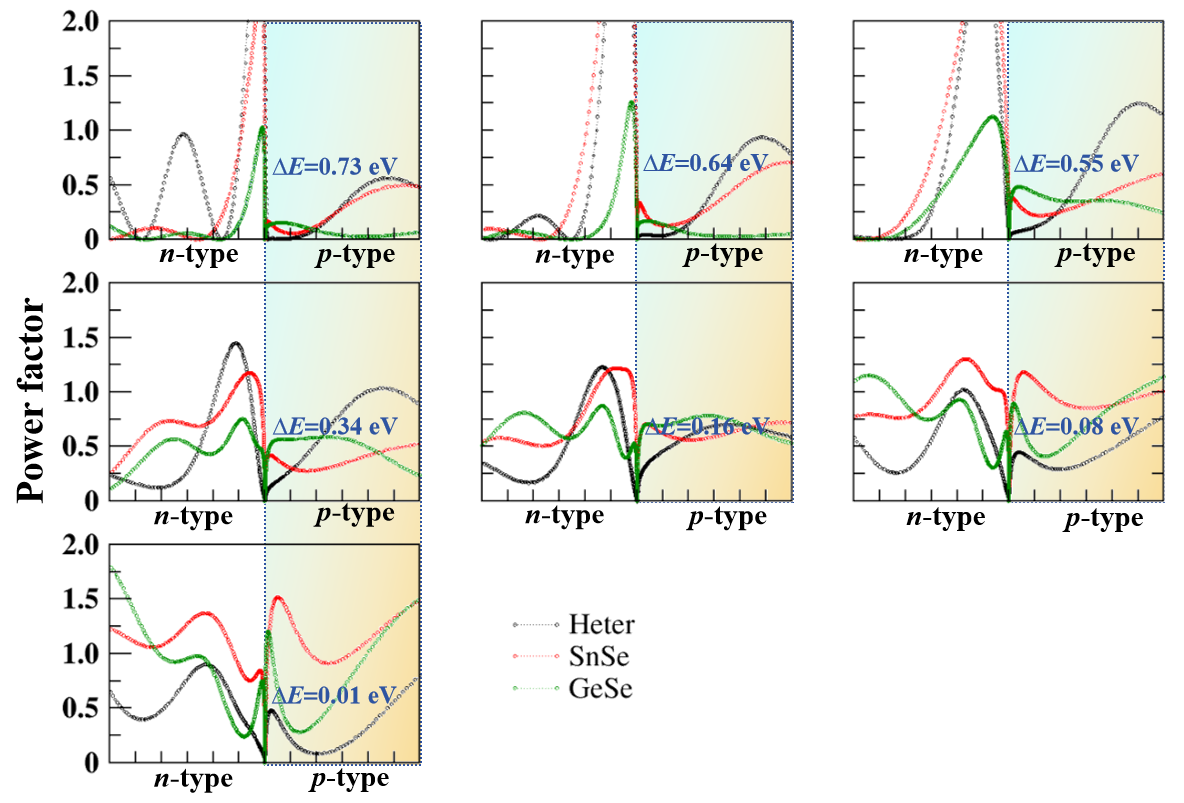


**Figure S4.** Calculated power factors along the *Γ*-*Z* direction. Calculated power factors of SnSe/GeSe superlattices and SnSe/GeSe supercells along the *Γ*-*Z* direction under interface-engineered valence band offsets. Power factor values are expressed in units of *τ*⁻¹10¹¹ W·m⁻¹·K⁻².


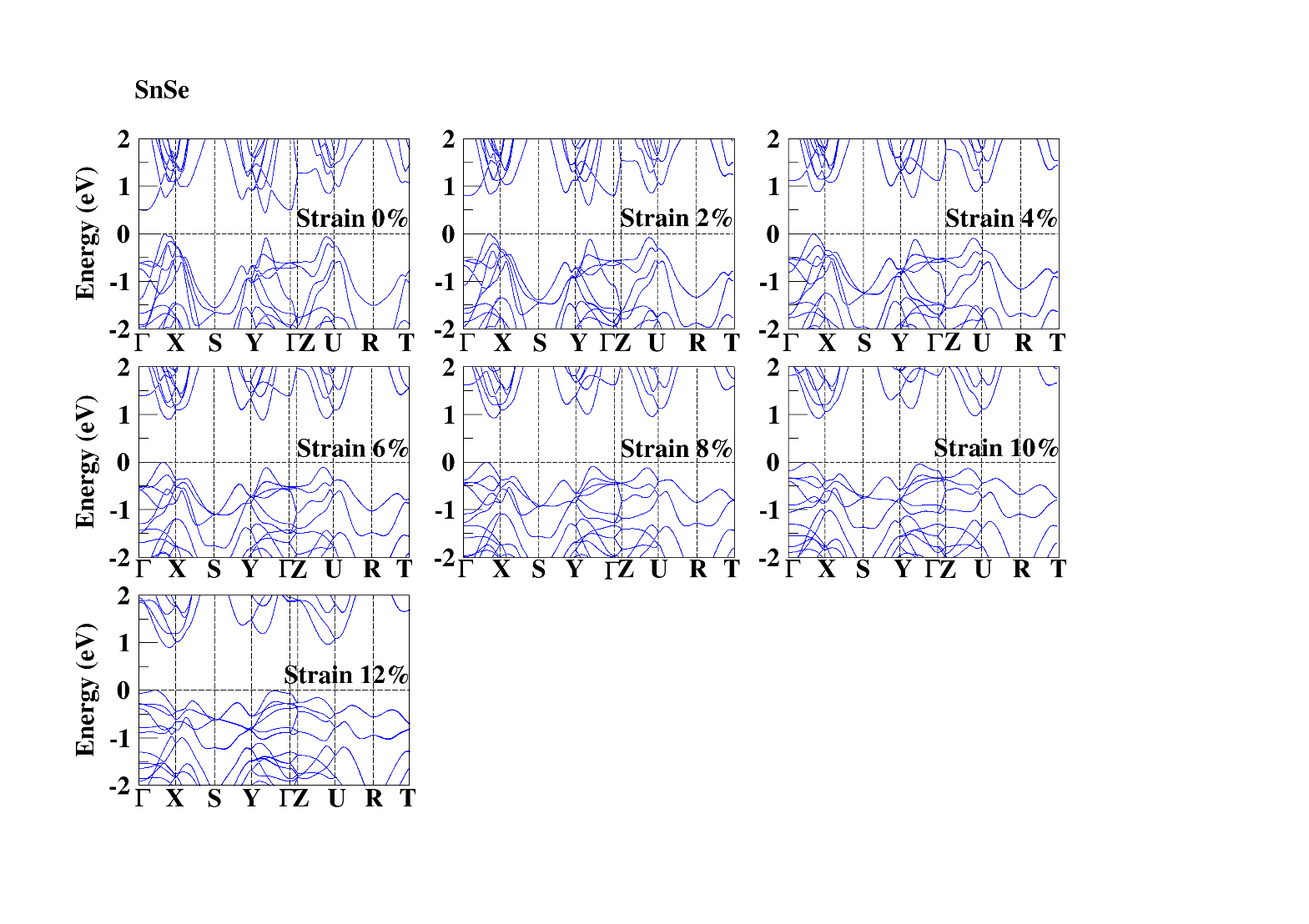


**Figure S5.** Electronic band structures of 1×1×4 SnSe supercells. Electronic band structures of the 1×1×4 SnSe supercell as a reference under conditions of continuously varying strain.


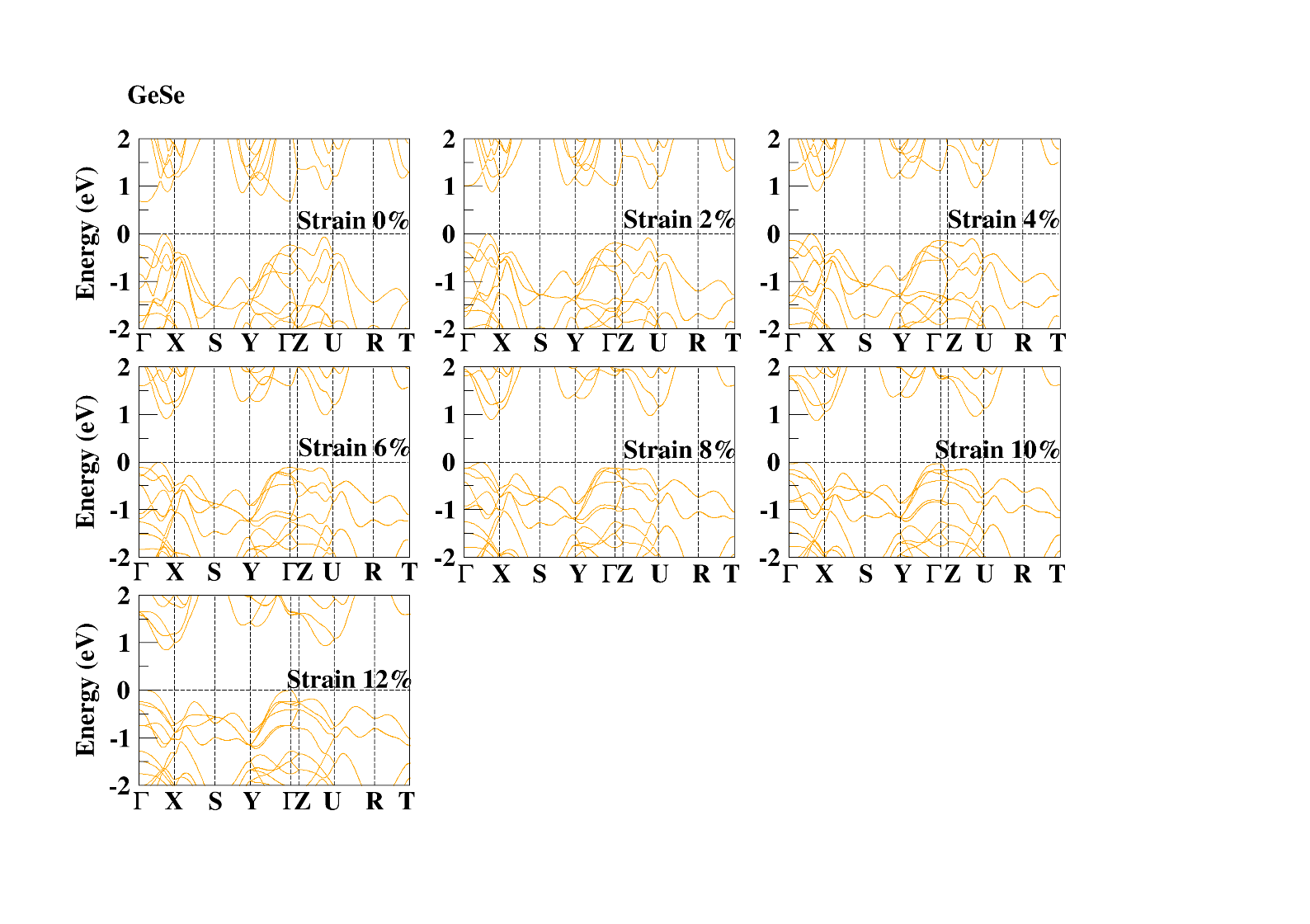


**Figure S6.** Electronic band structures of 1×1×4 GeSe supercells. Electronic band structures of the 1×1×4 GeSe supercell as a reference under conditions of continuously varying strain.

**_
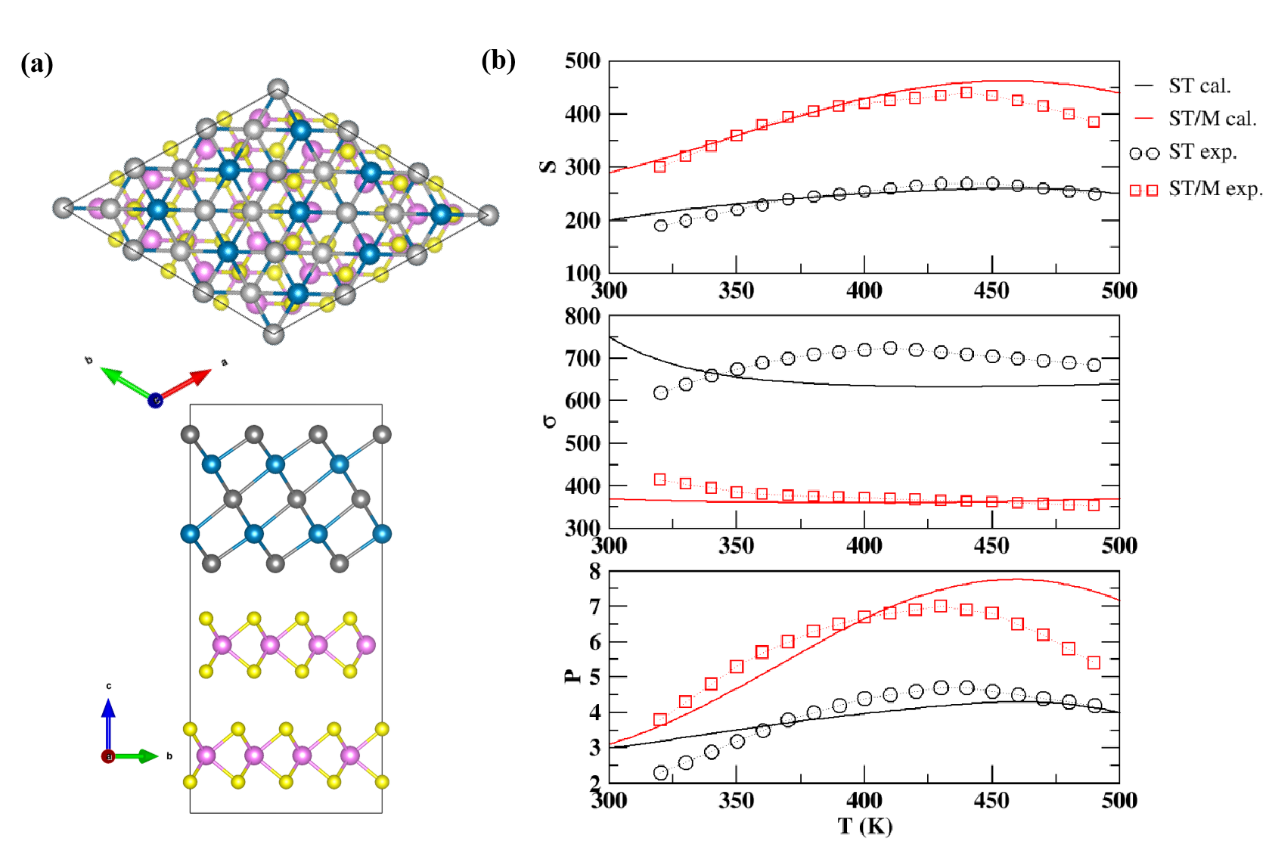
_**

**Figure S7**. (a) Structure models of Sb₂Te₃/MoS₂ heterostructure. (b) A comparison of experimental and theoretical thermoelectric parameters for Sb₂Te₃/MoS₂ heterostructures under the experimental valence band offset. A fixed carrier concentration, determined from experimental measurements, was used in the theoretical calculations to ensure consistency with the experimental conditions. Here, *S* is measured in μV/K, *σ* is in 10^2^ Ω^-1^m^-1^, and *P* is in 10^-3^ Wm^-1^K^-2^, respectively.


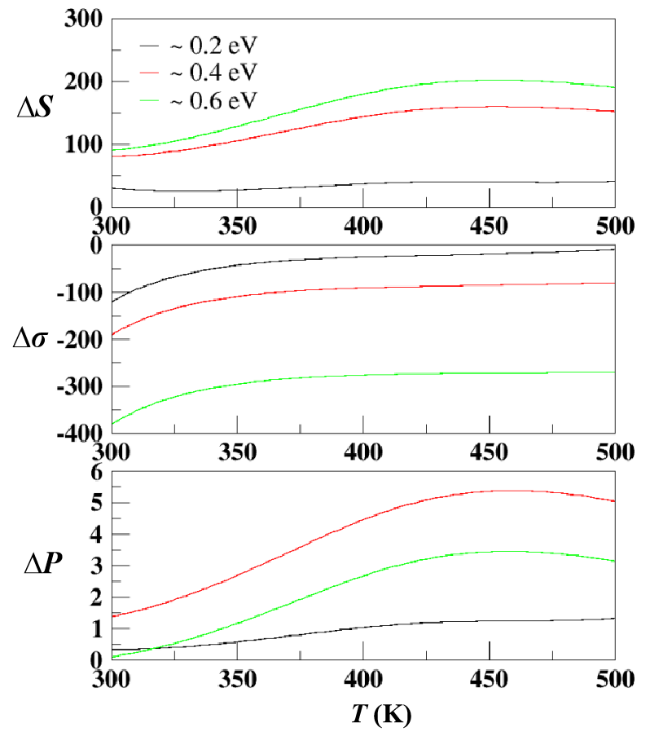


**Figure S8.** Temperature-dependent evolution of the additional thermoelectric parameters for Sb₂Te₃/MoS₂ heterostructures under three distinct strain-induced band offsets. Here, Δ*S* is measured in μV/K, ∆*σ* is in 10^2^ Ω^-1^m^-1^, and Δ*P* is in 10^-3^ Wm^-1^K^-2^, respectively.


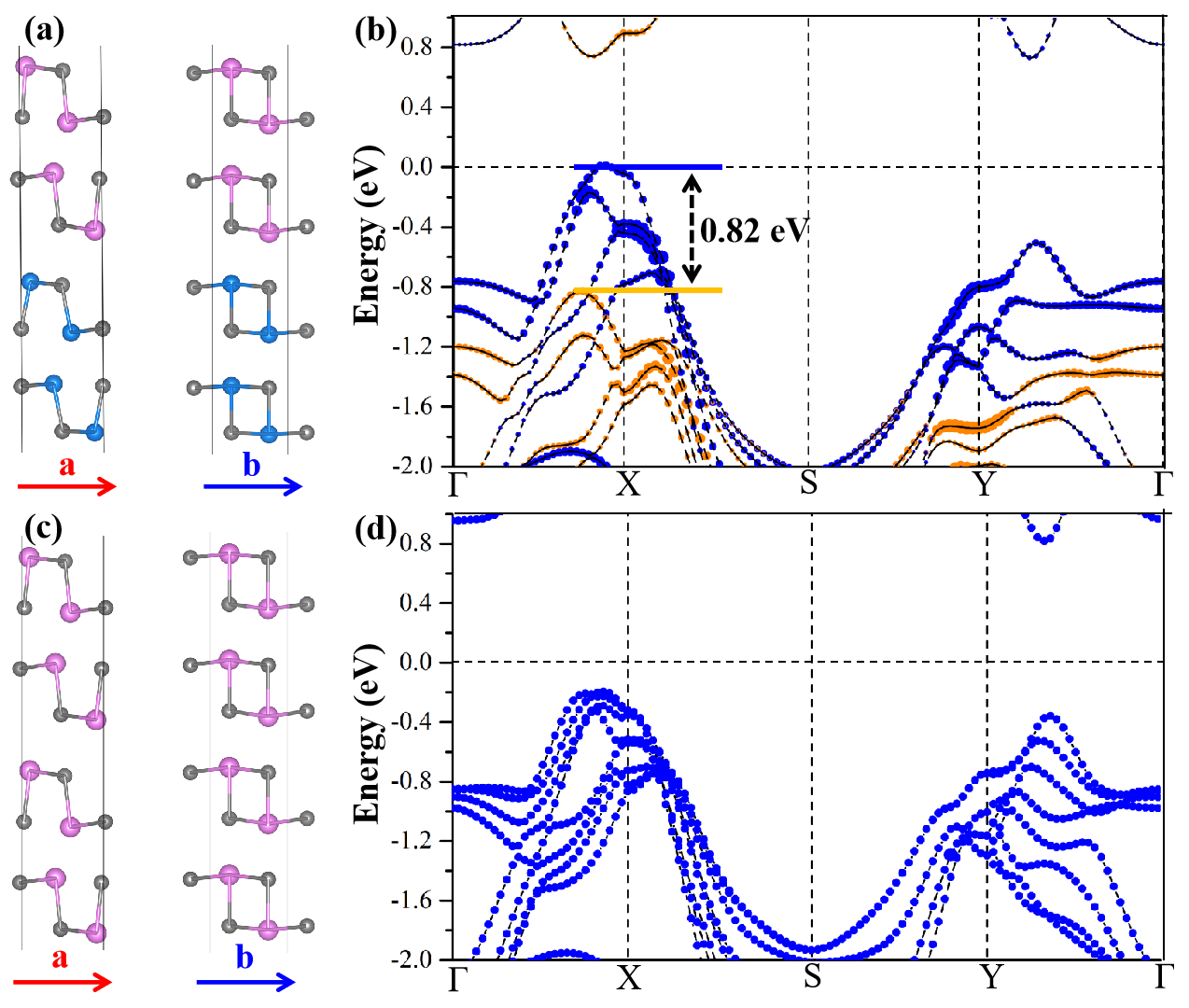


**Fig****ure S9.** Structure models and their electronic band structures. (a, c) Side views of SnSe/GeSe heterostructure and SnSe four-layer along x- (a-) and y- (b-) direction (axis). The pink, light blue and gray spheres represent Sn, Ge, and Se atoms, respectively. (b, d) Electronic band structures of SnSe/GeSe heterostructure and SnSe four-layer. The dark blue and orange dots represent the SnSe and GeSe components, respectively.

**Table S1.** Interlayer distance at heterointerfaces. Interlayer distance at heterointerfaces in SnSe/GeSe superlattices under different strains.

| **Strain** | **0%** | **2%** | **4%** | **6%** | **8%** | **10%** | **12%** |
| --- | --- | --- | --- | --- | --- | --- | --- |
| **Interlayer distance (Å)** | 3.08 | 3.06 | 3.09 | 3.04 | 3.06 | 3.05 | 3.10 |

**Table S2**. Experimental vs. DFT-calculated band offsets for representative heterostructures

| Heterointerface (A/B) | Experimental probe(s) | Exp. Δ*E*_v_/Δ*E*_c_ | DFT | Cal. Δ*E*_v_/Δ*E*_c_ | \|Δ\| (DFT – exp) |
| --- | --- | --- | --- | --- | --- |
| GeS/GeSe ^[1]^ | UPS + XPS | +0.23 / −0.16 | HSE06+ lineup | +0.21/−0.15 | ≤ 0.02 |
| GeS/GeSe ^[2]^ | HAXPES + XPS (IP) | Δ*E*_v_≈ +0.26 | HSE06 | ≈ +0.26 | ≈ 0 |
| SnS/SnSe ^[3]^ | ARPES (k-resolved) + XPS | +0.24/-0.14 | PBE & HSE06 | +0.26 / –0.14 | ≈ 0.02 |
| SnSe/SnSe₂  (vdW films) ^[4]^ | UPS | ≈ +1.0 / ≈ 0 | PBE | ≈ +0.9/ ≈ 0 | ≈ 0.1 |
| MoTe₂/SnSe₂ (2D/2D) ^[5]^ | KPFM + I_d_-V_g_ | Δ*E*_v_≈ 0.4 | PBE | ≈ 0.50 | 0.08 |

**References**

[1] M. Grodzicki, A. K. Tołłoczko, D. Majchrzak, D. Hommel, R. Kudrawiec, *Crystals* **2022**, 12, 1492.

[2] M. J. Smiles, J. M. Skelton, H. Shiel, et al., *J. Mater. Chem. A* **2021**, 9, 22440.

[3] A. K. Tołłoczko, S. J. Zelewski, J. Ziembicki, et al., *Adv. Opt. Mater.* **2024**, 12, 2302049.

[4] Y. Zhong, L. Zhang, W. Chen, H. Zhu, *Nanotechnology* **2020**, 31, 385203.

[5] F. Zhang, H. Shi, Y. Yali, et al., *Adv. Opt. Mater.* **2024**, 13, 2303088.
